# Research hotspots and global trends in respiratory syncytial virus over past five years

**DOI:** 10.3389/fmicb.2025.1599093

**Published:** 2025-08-04

**Authors:** Xiaoli Tao, Zhuping Ma, Hongxia Yuan, Wei Zhao, Jingyu Liu, Jing Tian

**Affiliations:** ^1^Department of Immunity and Pathogenic Microbiology, Jinzhou Medical University, Jinzhou City, China; ^2^Collaborative Innovation Center for Prevention and Control of Zoonoses, Jinzhou Medical University, Jinzhou, China; ^3^Division of Infectious Diseases, The First Affiliated Hospital of Jinzhou Medical University, Jinzhou City, China

**Keywords:** bibliometrics, trends, research hotspots, respiratory syncytial virus, VOSviewer

## Abstract

Respiratory syncytial virus (RSV) is the main cause of acute lower respiratory tract infections in children under 2 years old. This bibliometric analysis is used to determine the characteristics, hotspots, and frontiers of RSV global scientific output over the past 5 years. In this study, the Science Citation Index Expanded (SCI-Expanded) version from the Web of Science Core Collection (WoSCC) for publications and record information published from 2020 to 2024 was retrieved. Bibliometric software package was used to analyze the bibliometric indicators, and the research trends and hotspots of RSV were visualized by VOSviewer and Citespace. We assessed paper influence with the Global Citation Score (GCS). A total of 7,238 articles and comments were searched. The USA is the most productive country in the field of RSV research and also the country with the closest cooperation with other countries and institutions. The most influential journal in this field is “VIRUSES BASEL” with 246 publications. The co-citation analysis of references showed that the RSV-related topics with the highest focus are “covid-19 pandemic,” “respiratory syncytial virus prefusion,” “American academy,” and “protein vaccine.” From 2020 to 2024, keyword cluster and keyword burst analyses showed that “Respiratory Syncytial Virus,” “Infection,” and “Children.” “Viral co-infection,” “anti-virus,” and “vaccines” are currently research hotspots. The research area in this field is mainly distributed among “Immunology,” “Pediatrics,” “Pharmacology Pharmacy,” and “Biochemistry Molecular Biology.” Our study highlights the trends and hotspots in the field of RSV research over the past 5 years. Identifying the most critical indicators in the field of RSV research would be able to help researchers in this field better understand RSV and make decisions.

## Introduction

Respiratory syncytial virus (RSV) is the main viral pathogen causing acute lower respiratory tract infections (ALRTIs) RSV infection is common in children under 2 years old, and more common in children aged 6 weeks to 6months (Oumei et al., 2018; Liu et al., 2016; Rima et al., 2017). In addition, RSV is prone to infecting elderly individuals with weakened immunity. The two subtypes of RSV, namely, RSV-A and RSV-B, can cause infections throughout the season and are prevalent every year (Kim et al., 2021). RSV is easily transmitted through respiratory droplets, and the clinical manifestations of virus infection are mostly upper respiratory diseases, which can further develop into lower respiratory diseases (Paramore et al., 2004). In ordinary adults, RSV infection can cause mild symptoms similar to the common cold, such as runny nose, cough, and fever, but in children under 2 years old and adults over 65 years old, RSV infection may lead to lower respiratory tract transmission and more severe symptoms, such as coughing, breathing difficulties, eating difficulties, hypoxemia, and wheezing (Mizuta et al., 2013; van den Hoogen et al., 2003). This infection can lead to more severe pathology, including bronchiolitis and pneumonia. In addition, an important feature of RSV is to reinfect healthy children and adults previously infected with the virus (Cane, 2001; O'Shea et al., 2005). According to reports, RSV can cause nearly 33 million cases of ALRTIs in infants annually, of which 3.2 million require hospitalization (Shi et al., 2017). The Center for Disease Control and Prevention estimates that RSV has resulted in hospitalizations for over 60,000 children under 5 years old and 180,000 adults over 65 years; it has also resulted in deaths for up to 500 children and 14,000 elderly (McLaughlin et al., 2022; Branche et al., 2022). Considering the number of unconfirmed or unreported cases and the global hospitalization and mortality rates of RSV infection, these numbers may be higher (Mazur et al., 2023). Although RSV was first discovered over 60 years ago and has been widely studied since its discovery, no effective treatment for RSV infectious diseases is available at present. Therefore, the quantitative analysis of the current situation, areas of focus, and prospects of RSV is of great importance.

Bibliometrics is an interdisciplinary discipline that has been used in medicine and various other disciplinary fields (Qiu et al., 2018; He et al., 2021). Bibliometrics focuses on the systematic, bibliometric characteristics of literature and uses quantitative research methods to analyze the distribution, relationships, changes, and progress of literature in a certain field (Mao et al., 2018; Yang et al., 2023). Based on bibliometric analysis and visualization, the development trends and hotspots in this field can be understood. Our research may help improve the understanding of important nodes in the trends in this field by using Local Citation Score as an index. In this study, bibliometric analysis was used to analyze the research hotspots, keywords, references, journals, researchers, and research areas in the field of RSV from 2020 to 2024. However, this study includes only the Science Citation Index (SCI) Expanded versions of English articles and reviews, and is unable to analyze the full text of publications, so this work may have some limitations.

## Methods

### Sources of information and search methodologies

The literature related to RSV from 2020 to 2024 was compiled using the SCI-Expanded version of the Web of Science Core Collection (WoSCC) database. The search terms were as follows: TS = (“Respiratory syncytial virus” OR “Syncytial Virus” OR “syncytial-virus” OR “syncytial virus” OR “RSV” OR “RS Virus” OR “orthopneumovirus”) NOT TS = (“Rous sarcoma virus” OR “RSV oncovirus” OR “Ranikhet disease virus”). The operator “OR” can search for records containing any search terms separated by this operator, and the operator “NOT” can exclude items that interfere with the exactitude of our search results. Double quotation marks can ensure that these words are searched as a whole. In various types of publications, only English-language articles and reviews were considered. A total of 7,290 articles were ultimately analyzed from 2020 to 2024.

### Data collection and cleaning

The recorded information includes the number of papers and citations, H-index, publication year, countries/regions, affiliations, authors, journals, references, and keywords. Then, duplicate authors and misspelled elements were manually removed. Although inaccurate analysis may not be completely avoided due to multiple versions of cited literature, identical abbreviations of different authors, and different forms of cited journals, most of the raw data are reliable. Then, to analyze the data further, online programs,^1^ VOSviewer_v1.6.20, and CiteSpace (version 6.4. R1) were used.

### Bibliometric analysis

The number of publications (NP) and the number of citations (NC) commonly used to represent bibliographic materials are examples of bibliometric indicators. As two fundamental perspectives for measuring research performance, NP quantifies production capacity, and NC demonstrates its influence. The H-index evaluates the academic contributions of researchers and predicts future scientific achievements. The H-index links productivity and influence by determining a threshold that links NP and NC. However, it can now also define the publication output of a country or region and the output of an institution or journal (Wang et al., 2021; Noruzi et al., 2022). In addition, the impact factor (IF) calculated based on the latest version of the Journal Citation Report is widely regarded as one of the most important indicators of the quality and influence of medical journals (Roldan-Valadez et al., 2019). The Global Citation Score (GCS) is considered the NC of an article on a global scale. The GCS is an important indicator of the contribution of an article to the field of knowledge, and a high GCS indicates a high level of interest among scientists around the world (Khazaneha et al., 2021). VOSviewer is used to construct and visualize a bibliometric network diagram (Centre for Science and Technology Studies, Leiden University, Leiden, the Netherlands) (Deng et al., 2022; van Eck and Waltman, 2010).

VOSviewer was used for co-citation, co-occurrence and co-authorship analysis in this study. The size of the node represents the NPs, the thickness of the line represents the strength of the relationship, and the colors of the nodes represent different clusters or periods. In addition, cluster analysis, timeline or time zone views, references, and keyword citation bursts are analyzed by CiteSpace to aid in the visual evaluation of knowledge domains and development trends (Liu et al., 2021). Before data analysis by CiteSpace, the duplicates were removed. Cluster analysis can classify references and keywords, and identify important research topics for RSV. The explosion of keywords and references is often used to discover new research trends.

## Results

### Overview of publications on RSV

Through searching the search terms, a total of 21,546 papers were retrieved, a total of 7,238 articles and reviews in the past 5 years were retrieved from the SCI-extended version of WoSCC based on age, article type, and language limitations. The total NC of the retrieved articles was 91,185, and the average NC per article was 16.51. The H-index for all publications was 115. The results of the thorough screening are presented in Table 1.

**Table 1:** Flowchart of the screening process.

Set	Results	Refinement
1	21,546	TOPIC: (TS = ("Respiratory syncytial virus" OR "Syncytial Virus" OR "syncytial virus" OR "RSV" OR "RS Virus" OR "orthopneumovirus") NOT TS = ("Rous sarcoma virus” OR "RSV oncovirus")) Indexes = SCI-EXPANDED
2	8,333	Refined by PUBLICATION YEARS: (2020 OR 2021 OR 2022 OR 2023 OR 2024)
3	7,290	Refined by DOCUMENT TYPES: (ARTICLES OR REVIEW ARTICLES)
4	7,238	Refined by LANGUAGES: (ENGLISH)

### Annual trend in the quantity of paper publication

**Figure 1:**
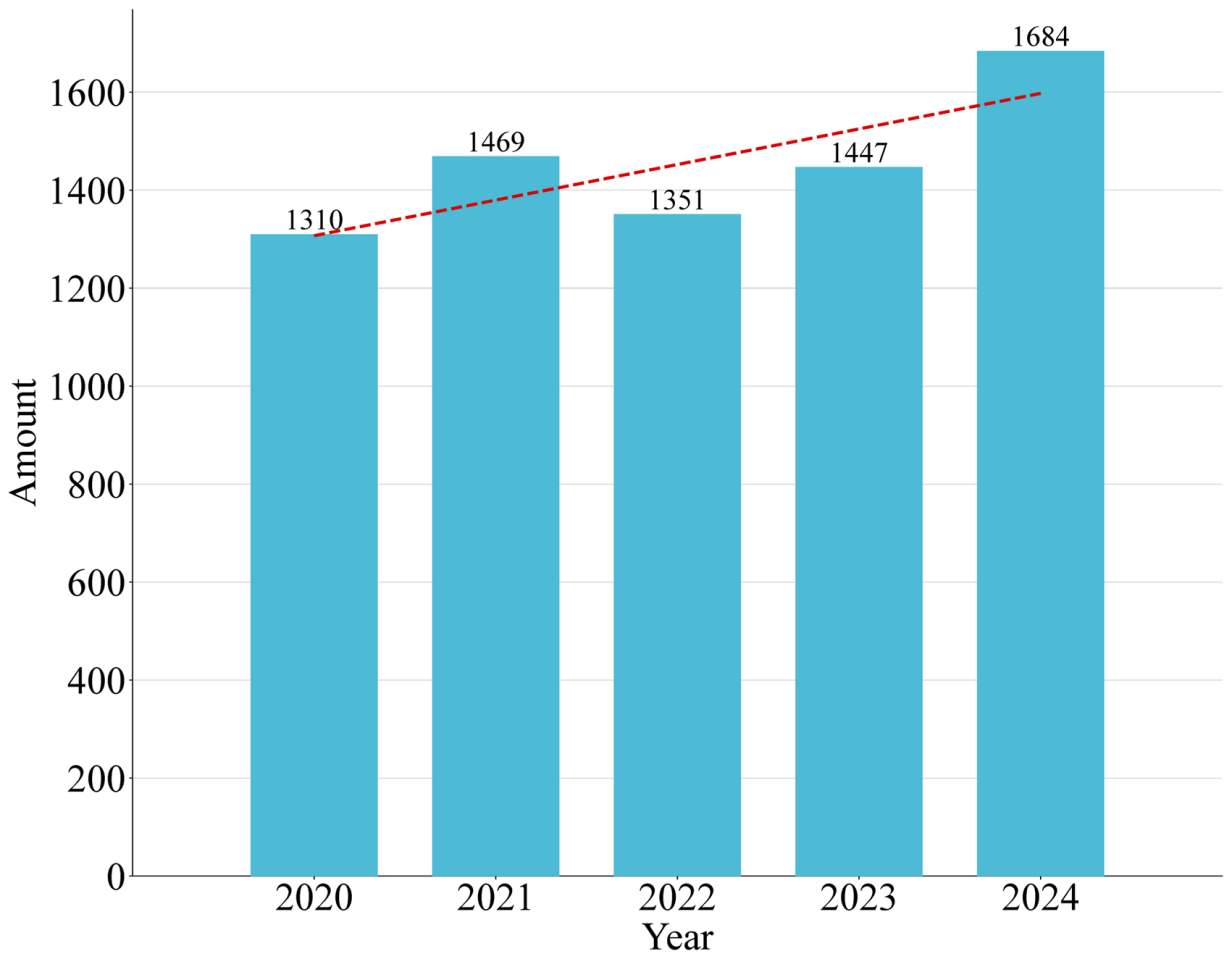
Amount of publications by year during the past 5 years.

Figure 1 displays yearly NPs as it relates to RSV. The number of yearly papers increased from 1,319 in 2020 to 1,684 in 2024. Despite minor fluctuations in the past 5 years, more articles were published overall, indicating that although the NPs of RSV research showed an increasing trend in the past 5 years, it is not stable. The possible reason for this unstable trend is the impact of the COVID-19 epidemic. On the whole, from 2020 to 2024, the NPs in this field increased obviously, with slight fluctuations during this period. The five year growth rate was calculated as: (2024 value/2020 value) × (1/5) = 25.53%.

### Contributions of countries/regions to global publications

**Figure 2:**
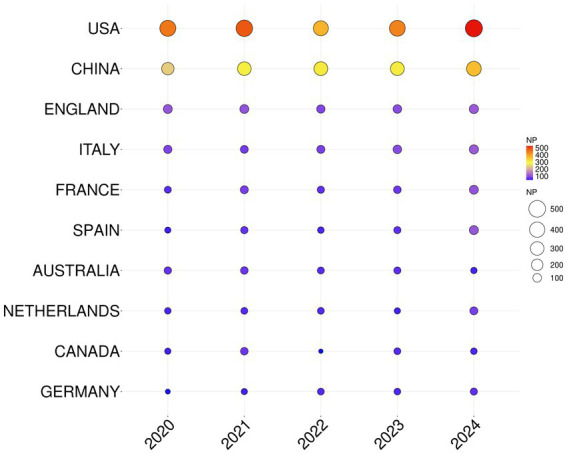
Top 10 countries/regions in terms of annual publications on RSV research from 2020 to 2024. The circle’s size and colors show the number of papers. The larger the circle, the color from blue to red, the higher the NP issued in that country.

Countries/regions analyses showed that the 7,238 articles were from 147 countries/regions. Figure 2 shows the top 10 countries/regions in terms of annual publication on RSV research from 2020 to 2024. Table 2 ranks the top 10 high-output countries/regions of all authors according to NPs. The annual publication data of these 10 countries were also analyzed (Figure 2, Supplementary material S1). The USA published the most articles (2,278/33.1%), followed by China (1,524/22.14%) and England (527/7.65%). Papers from the USA were cited 44,846 times, accounting for 31.61% of total citations, followed by China (21,101/14.87%) and England (14,915/10.51%). However, in 2022, NPs has shown a downward trend in the USA, whereas it has been steadily increasing in China. This development indicates that more Chinese researchers are paying attention to RSV research now. Additionally, the USA reached the highest H-index (89), much higher than that of other countries/regions. In the top 10 productive countries/regions, Australia and England had higher average citation per item but lower NPs and H-index.

**Table 2:** Top 10 countries with the highest productivity.

Rank	Countries/regions	NP	NC	H-index	Average citation per item
1	USA	2,278	44,846	89	22.34
2	China	1,524	21,101	59	15.04
3	England	527	14,915	57	29.68
4	Italy	475	9,635	46	21.97
5	France	410	8,892	49	23.07
6	Spain	372	8,837	44	25.4
7	Australia	347	10,168	47	30.41
8	Netherlands	335	8,741	48	27.97
9	Canada	312	7,181	42	23.75
10	Germany	305	7,548	44	25.85

**Figure 3:**
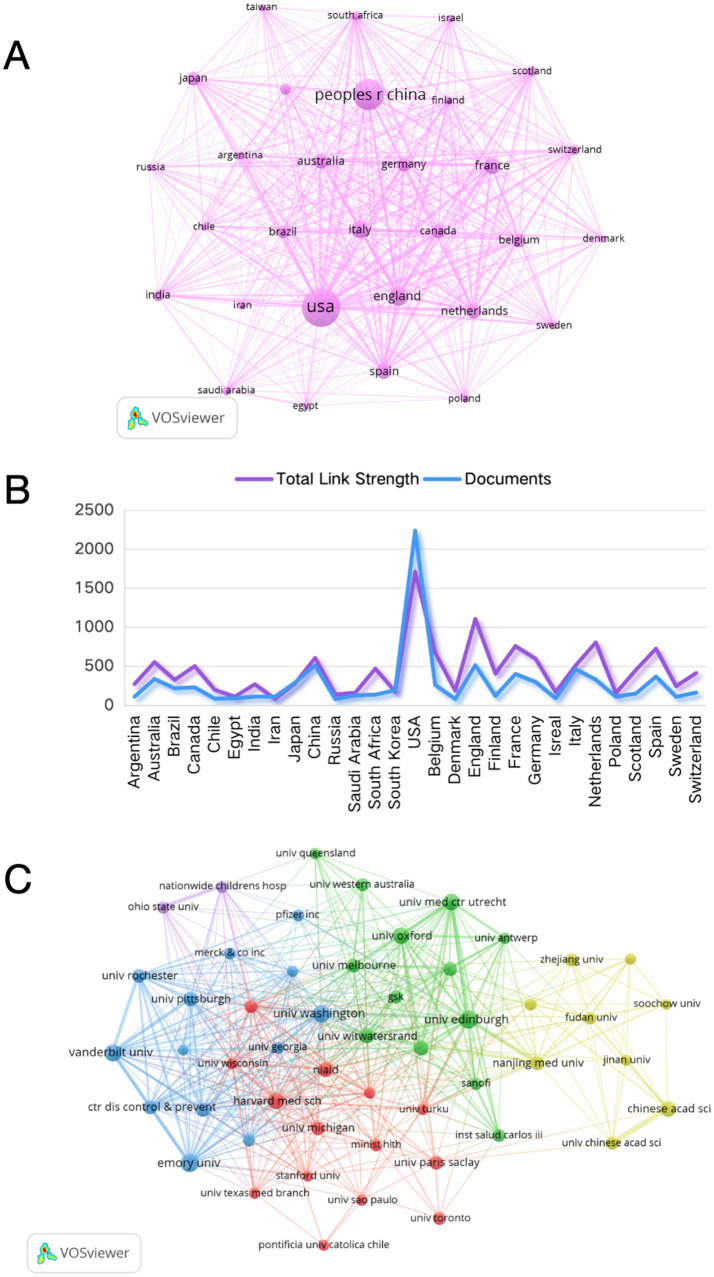
The collaborative network of countries/regions and affiliations. **(A)** Collaboration network of the top 30 most productive countries/regions. **(B)** The total link strength and documents of cooperation among the productive countries/regions. **(C)** Collaboration network of the top 50 affiliations.

Effective cooperation between institutions and countries plays an important role in promoting academic exchange and scientific research development. Figure 3A shows the collaborative networks of the top 30 countries/regions with the highest number of publications in this field from 2020 to 2024. Among them, the USA and China are the two major producers of research in this field, and their cooperation far exceeds that of other countries. Secondly, the USA has relatively frequent cooperation with countries such as the United Kingdom, Canada, and Italy, while China has also shown close cooperation and exchanges with Australia, Germany, and France.

Figure 3B demonstrates the total link strength and documents of cooperation among the top 30 most productive countries, reflecting the scale of international cooperation and, to some extent, revealing the hub status and degree of dependence of countries within the research network. The analysis revealed that the USA had a higher documents (2,236) than its total link strength (1,711), indicating that USA possessed relatively complete independent research capabilities and scientific research infrastructure support in the field of RSV. Countries such as England (total link strength 1,105, documents 514), the Netherlands (total link strength 804, documents 328), and Spain (total link strength 725, documents 367) exhibited a phenomenon where the total link strength exceeded the number of documents. This suggested that these countries may rely more on international cooperation networks to drive the development of RSV research, and their scientific research systems may have a stronger ability to integrate international resources or specific patterns of specialized division of labor and cooperation in certain fields.

### Analysis of affiliations

Table 3 displays the top 10 affiliations with the highest NPs related to RSV. “Institut national de la santé et de la recherche médicale (INSERM)” in France has the highest NP (182), followed by two American institutions: “Harvard University” (168) and “Centers for Disease Control Prevention USA” (142). It is worth noting that the “University of Edinburgh,” ranked ninth, performed the best, with the highest having NC (4,827), H-index (37) and average citation per item (41.1), indicating that its articles have a high level of influence in the field of RSV. In addition, the top 10 affiliations included seven American affiliations, two English institutions, and one French affiliation. The findings indicate that American institutions have high research capabilities and academic standards in the field of RSV research. However, China, which ranks second in terms of NP, has not been listed by any research affiliation. In addition, we also analyzed the cooperative relationships between research institutions (Figure 3C), and the results showed that the University of Edinburgh maintains close cooperative relationships with University Medical Center Utrecht and Nanjing Medical University.

**Table 3:** Top 10 most productive affiliations.

Rank	Affiliations	Countries/Regions	NP	NC	H-index	Average citation per item
1	Institut national de la santé et de la recherche médicale (INSERM)	France	182	3,758	30	21.4
2	Harvard University	USA	168	4,611	36	28.33
3	Centers for Disease Control Prevention USA	USA	142	4,392	28	32.14
4	University of London	England	137	2,979	26	22.54
5	University of California System	USA	134	3,562	31	26.96
6	University System of Ohio	USA	134	3,095	29	23.72
7	Harvard University Medical Affiliates	USA	130	3,266	29	26.04
8	National Institutes of Health NIH USA	USA	124	4,155	32	34.47
9	University of Edinburgh	England	124	4,827	37	41.1
10	Johns Hopkins University	USA	122	4,002	30	33.46

### Analysis of authors

The top 10 productive authors are listed in Table 4. They contributed 405 publications, accounting for 5.6% of the total number of articles in the past 5 years. Nair, Harish (56), from the University of Edinburgh, tied for the first place in the field of studying on RSV, followed by Bont, Louis J. (45) from the Utrecht University, and Martinon-Torres, Federico (43) from the Complexo Hospitalario Universitario de Santiago de Compostela. It is interesting that although Bont, Louis J., who ranks second, has a higher number of articles, he is relatively low in terms of NC (883), H-index (17), and average citation per item (20.71). It is noted that Madhi, Shabir, ranked fourth, from University of Witwatersrand in South Africa, has the highest average citation per item (95.73) for their articles. It indicates that his research results have been recognized by many researchers. Furthermore, the top 10 authors with the highest number of publications from 7 countries/regions, which indicates that many researchers around the world are dedicated to RSV research.

**Table 4:** Top 10 authors with the most publications.

Rank	Author	NP	NC	Countries/Regions	Affiliation	H-index	Average citation per item
1	Nair, Harish	56	3,447	Scotland	University of Edinburgh	28	63.64
2	Bont, Louis J.	45	883	Netherlands	Utrecht University	17	20.71
3	Martinon-Torres, Federico	43	1,191	Spain	Complexo Hospitalario Universitario de Santiago de Compostela	18	28.86
4	Madhi, Shabir	40	3,757	South Africa	University of Witwatersrand	19	95.73
5	Bont, L. J.	39	1,411	Netherlands	Utrecht University	18	36.87
6	Atwell, Jessica E.	37	2,882	USA	University of Colorado Medical Campus	19	79.84
7	Piedra, Pedro A.	37	1,942	USA	Baylor College of Medicine	16	53.49
8	Eléouët, Jean-François	37	655	France	INRAE	12	19.08
9	Li, You	36	2,246	Scotland	University of Edinburgh	19	64.83
10	Kalergis, Alexis M.	35	559	Chile	Pontificia Universidad Catolica de Chile	15	18.69

### Analysis of journals

Table 5 presents the top 10 most published Journals that in the past 5 years, Viruses-Basel published the most studies on RSV (246 publications, IF (2024): 3.5), followed by the Journal of Infectious Diseases (188 publications, IF (2024): 4.5) and Frontiers in Immunology (161 publications, IF (2024): 5.9). Viruses-Basel encourages scientists to publish their experimental and theoretical results in as much detail as possible, fosters the publication of timely reviews and commentaries on topics of interest to the virology community, and features highlights from the virology literature in the “News and Views” section. Most of the top 10 journals had a higher IF (defined as >3.000). Among the 10 journals, although the Journal of Infectious Diseases, ranked second, has slightly lower NP, its NC (4,019), H-index (34), and average citation per item (22.9) are much higher than the first one. In addition, approximately 19.23% of the papers were published in the top 10 academic journals (1,392/7,238).

**Table 5:** Top 10 most-published journals.

Rank	Journal	NP	NC	IF(2024)	H-index	Average citation per item
1	Viruses Basel	246	3,130	3.5	25	13.2
2	Journal of Infectious Diseases	188	4,019	4.5	34	22.9
3	Frontiers in Immunology	161	3,594	5.9	30	22.68
4	Influenza and Other Respiratory Viruses	144	2,046	4.2	23	14.8
5	PLOS One	126	1,097	2.6	16	8.82
6	Scientific Reports	113	1,502	3.9	20	13.36
7	Vaccines	107	1,191	3.4	19	11.79
8	Vaccine	103	1,186	3.5	19	11.86
9	International Journal of Molecular Sciences	102	1,543	4.9	21	15.28
10	Journal of Virology	102	1,408	4.6	19	14.08

### Analysis of paper GCS

**Figure 4:**
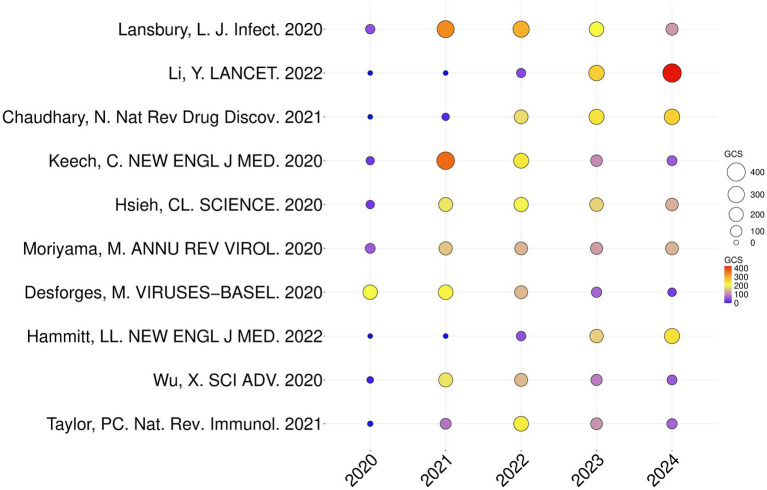
Yearly number of global citations for papers having a high GCS. The circle’s size and colors show the GCS of papers. The larger the circle and the color from blue to red, the higher the GCS of the article and the more influential it is in the field.

The number of citations in an article can reflect the hotspots and trends of RSV research. Table 6 lists the top 10 most cited papers in this field, and Figure 4 presents the number of GCS per year for the most influential top 10 papers. The GCS of the paper written by Lansbury, L. J. et al. in 2020 was 1,013, ranking first. In this review article, the authors assessed the burden of patients with Corona Virus Disease 2019 (COVID-19) who are infected with other pathogenic microorganisms, including RSV and influenza virus (Lansbury et al., 2020). The GCS of another article, which was published in *Lancet* (IF: 88.5) and written by Li. Y. et al. in 2022, was 728. The authors systematically analyzed the global, regional, and national disease burden of acute lower respiratory tract infections caused by respiratory syncytial virus in children under 5 years old in 2019 (Li et al., 2022). The fifth-ranked article was published by Chaudhary, N. et al. in 2021 in Nat. Rev. Drug Discov., which has a high impact factor. It not only described the basic technology of mRNA vaccines, with a focus on lipid nanoparticles and other non-viral delivery carriers, but also outlined mRNA vaccines targeting various infectious disease pathogens (Chaudhary et al., 2021). In addition, most of these highly cited papers were about the research of COVID-19 vaccine based on RSV (Keech et al., 2020; Hsieh et al., 2020; Taylor et al., 2021; Lee et al., 2020). Nevertheless, these documents had a substantial effect on the study of RSV, which increased the number of subsequent literature publications on the subject.

**Table 6:** The most-cited paper from 2020 to 2024 in the field of RSV.

Rank	Year	Article	IF (2024)	Total citation	Type of study
1	2020	Lansbury, L. et al. Co-infections in people with COVID-19: a systematic review and meta-analysis. *J. Infect*. doi: 10.1016/j.jinf.2020.05.046	11.9	1,013	Review
2	2020	Keech, C. et al. Phase 1–2 Trial of a SARS-CoV-2 Recombinant Spike Protein Nanoparticle Vaccine. *NEW ENGL J MED*. doi: 10.1056/NEJMoa2026920	78.5	797	Article
3	2020	Hsieh, CL. et al. Structure-based design of prefusion-stabilized SARS-CoV-2 spikes. *SCIENCE*. doi: 10.1126/science.abd0826	45.8	740	Article
4	2022	Li, Y. et al. Global, regional, and national disease burden estimates of acute lower respiratory infections due to respiratory syncytial virus in children younger than 5 years in 2019: a systematic analysis. *LANCET. doi:*10.1016/S0140-6736(22)00478-0	88.5	728	Review
5	2021	Chaudhary, N. et al. mRNA vaccines for infectious diseases: principles, delivery and clinical translation. *Nat. Rev. Drug Discov*. doi: 10.1016/S0140-6736(19)30721-4	101.8	697	Review
6	2020	Desforges, M. et al. Human Coronaviruses and Other Respiratory Viruses: Underestimated Opportunistic Pathogens of the Central Nervous System? *VIRUSES-BASEL*. doi:10.3390/v12010014	3.5	682	Review
7	2020	Moriyama, M. et al. Seasonality of Respiratory Viral Infections. *ANNU REV VIROL*. doi:10.1146/annurev-virology-012420-022445	8.3	634	Article
8	2020	Wu, X. et al. Air pollution and COVID-19 mortality in the United States: Strengths and limitations of an ecological regression analysis. *ADV SCI.* doi: 10.1126/sciadv.abd4049	12.5	502	Article
9	2021	Taylor, PC. et al. Neutralizing monoclonal antibodies for treatment of COVID-19. *Nat. Rev. Immunol.* doi: 10.1038/s41577-021-00542-x	60.9	493	Review
10	2022	Hammitt, LL. et al. Nirsevimab for Prevention of RSV in Healthy Late-Preterm and Term Infants. NEW *ENGL J MED.* doi: 10.1056/NEJMoa2110275	78.5	463	Article

**Figure 5:**
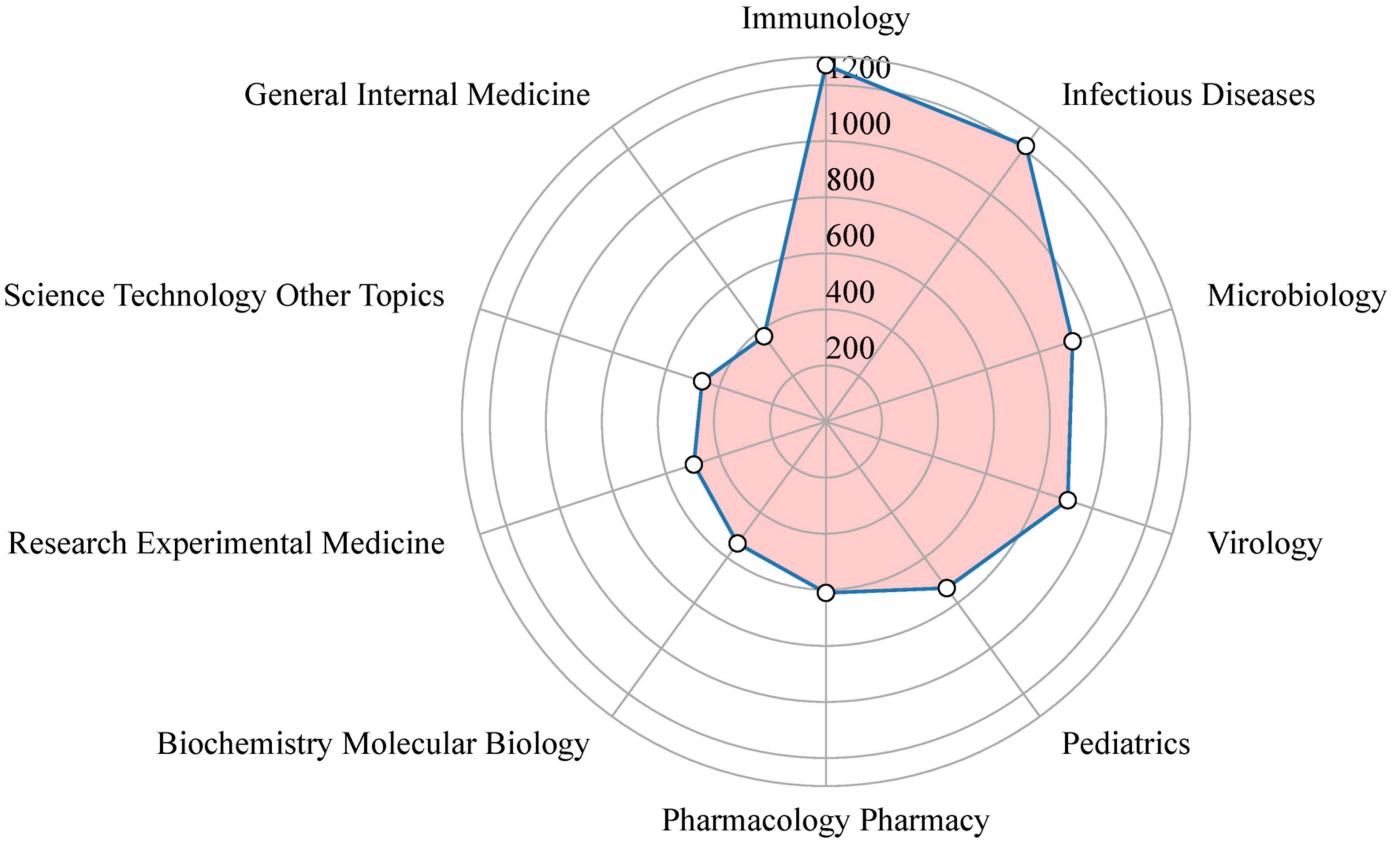
Radar map of the top 10 research productive categories on RSV.

Figure 5 displays the top 10 productive categories associated with RSV. The most prevalent study categories were “Immunology” (1,271 papers), “Infectious Diseases” (1,215 papers), “Microbiology” (926 papers), “Virology” (908 papers), “Pediatrics” (734 papers), “Pharmacology Pharmacy” (611 papers), “Biochemistry Molecular Biology” (537 papers), “Research Experimental Medicine” (496 papers), “Science Technology Other Topics” (465 papers), and “General Internal Medicine” (376 papers). “Immunology” had the highest proportion of publications in terms of research categories, whereas “General Internal Medicine” had the lowest proportion. We also counted the annual publication volume related to a certain research field (Supplementary material S2). The results show that research on “Immunology” has consistently maintained a high number in RSV studies, followed by “Infectious Diseases.”

### Analysis of co-cited references

**Figure 6:**
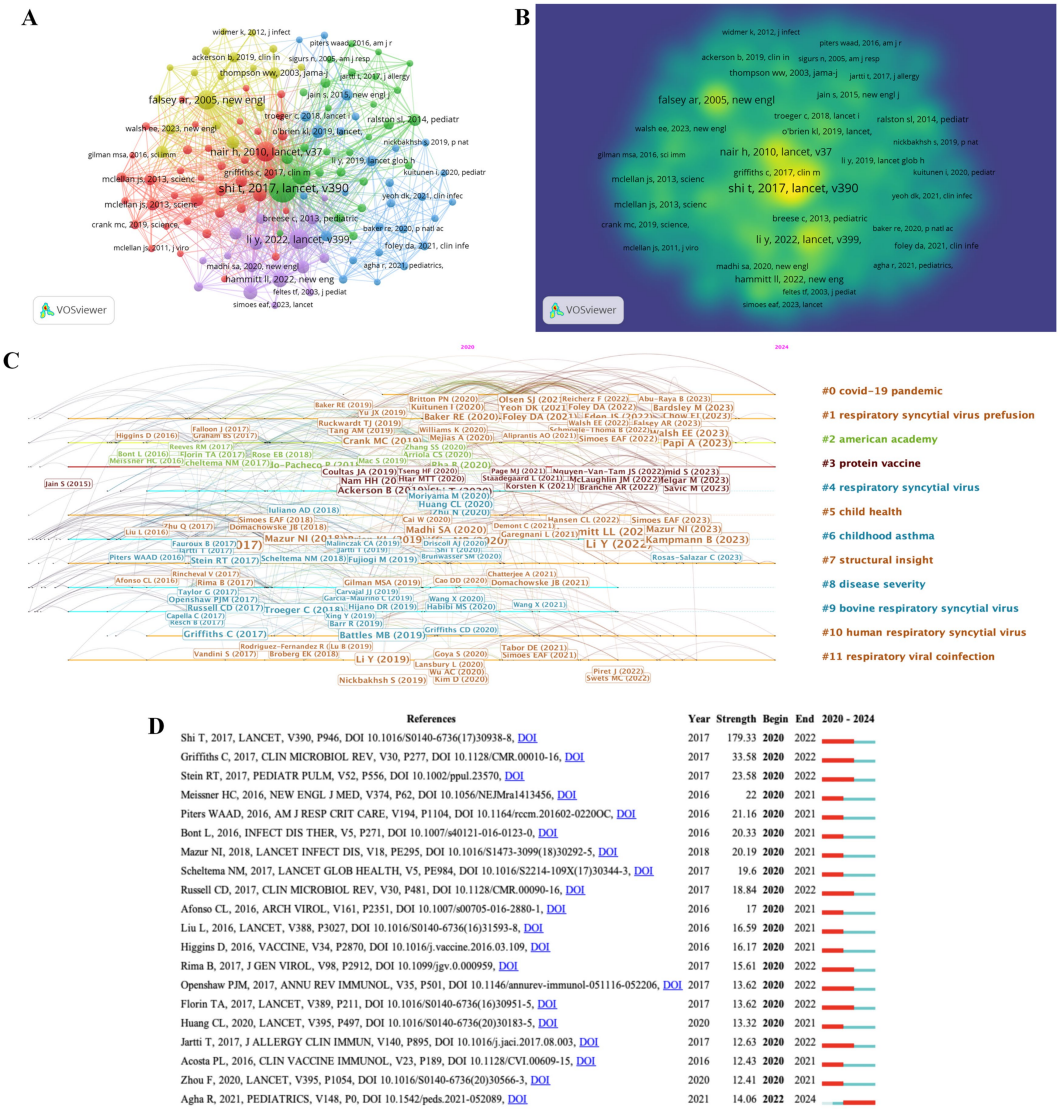
Mapping based on co-cited references from RSV-related research. **(A)** Network diagram of co-cited references. Of the 227,194 references, 128 (divided into 5 clusters) were cited at least 72 times. **(B)** Density visualization for 128 co-cited references network map. Each keyword in the density visualization has colors that indicates its density. Yellow means appearing more frequently, while green means appearing less frequently. **(C)** Top 20 co-cited references with the strongest citation bursts. The years between “Begin” and “End” represent the period when the reference was more influential. **(D)** Top 20 References with the Strongest Citation Bursts. Years in light green mean that the reference has not yet published, and years in dark green mean that the reference has a less influential. Instead, years in red represents that the reference has a higher influence.

Different from global citation analysis, the co-citation network stresses the research themes closely related to a specific field. Considering the masses of cited references, the minimum NC of a reference was set as 72. Of the 227,194 references cited by the retrieved papers, 128 references were selected for co-citation analysis, which was divided into four clusters (Figure 6A). The line connecting two nodes shows that both were cited in one publication, a shorter line represents a closer relationship between two papers, and a shorter line means a closer association between the two references. Moreover, the size of nodes stands for the total link strength, representing the total number of co-citations of a document. Different colors of nodes were employed to divide the references into 5 clusters: Cluster 1 (in red) included 38 items, which mainly studied the development and preventive effects of RSV vaccines. Cluster 2 (green) included 28 items, which focused the epidemic status and the national disease burden of RSV infection. Cluster 3 (blue, 27 items) centered on prevention and treatment strategies for the infection of RSV in young children. Cluster 4 (in yellow, 19 items) concentrated on the classification and genetic variability of RSV. Cluster 5 (in purple, 16 items) discussed the development of drugs for prevention of RSV infection. For further study of the co-citation of macrophage-function-related references, density visualization was used to analyze 33 references (Figure 6B). Density visualization often shows the overall structure of the study and highlights important research areas. Figure 6C indicates the most typical references in terms of burst length, burst strength, and burst time. The top 10 clusters of co-cited references were as follows: “covid-19 pandemic,” “respiratory syncytial virus prefusion,” “American academy,” “protein vaccine,” “respiratory syncytial virus,” “child health,” “childhood asthma,” “structure insight,” “disease severity,” and “bovine respiratory syncytial virus.” Among them, brown “protein vaccine” is currently the most popular research direction.

The top 20 references with the most powerful citation bursts are depicted in Figure 6D. The publication written by Shi T, et al. in 2017 had the highest strength (179.33) from 2020 to 2022, more than five times that of the second reference (Agha and Avner, 2021) (0.58). This article, which was published on the *LANCET*, evaluated the global, regional, and national disease burden of acute lower respiratory tract infections caused by respiratory syncytial virus in young children in 2015 using a modeling system (Shi et al., 2017). The article ranked second in strength value was written by Griffiths C. et al. (33.58). It discussed the development of understanding of RSV replication, pathogenesis, diagnosis, and treatment, and attempted to reconcile the vast amount of information about RSV, as well as analyze the reasons why there are still no effective RSV vaccines and few treatment methods available (Griffiths et al., 2017). In 2021, Agha R. et al. published a paper in Pediatrics, they found that due to the impact of the COVID-19, the number of RSV cases began to increase in spring and peaked in summer instead of typical autumn and winter, and the peak and infection rate of older children were higher than expected (Agha and Avner, 2021). The above-mentioned high-strength literature has conducted relevant research on RSV from a variety of perspectives, including epidemiology, prevention and treatment strategies, and development of antiviral drugs of RSV.

### Analysis of research hotspots

**Figure 7:**
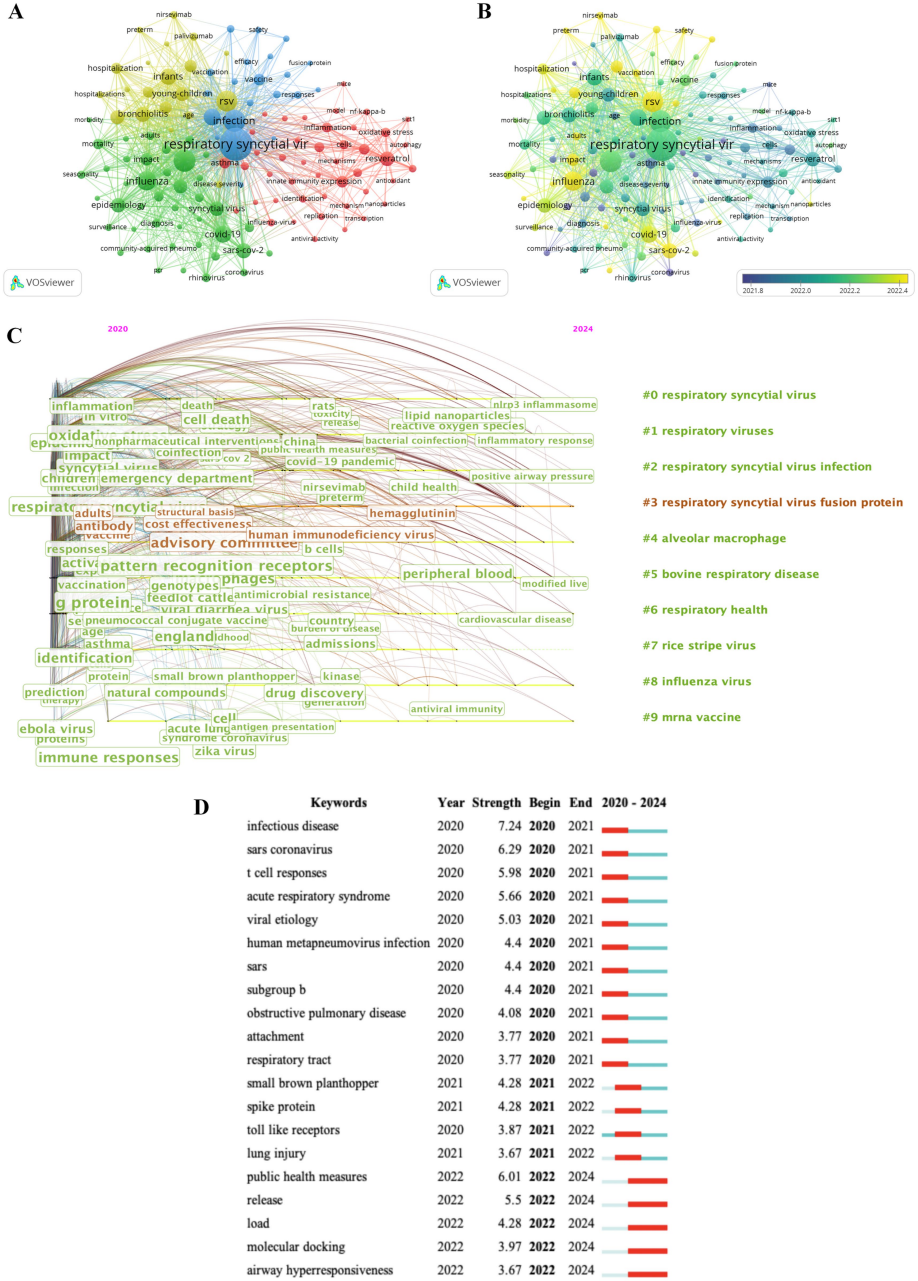
Keywords mapping of RSV research. **(A)** Of the 19,577 keywords, 127 terms that appeared more than 70 times were separated into 4 clusters by different colors: Cluster 1 is in red, Cluster 2 is in green, Cluster 3 is in blue, and Cluster 4 is in yellow. The size of the nodes indicates occurrence frequency. **(B)** Keyword visualization according to the APY. The different colors indicate the relevant year of publication. Yellow keywords came later than purple keywords. **(C)** Timeline distribution of keyword cluster analysis. **(D)** Top 20 keywords with the most bursts. The years between “Begin” and “End” represent the period when the keyword was more influential. Years in light green mean the keyword has not yet appeared, years in dark green mean the keyword has a less influence, and years in red represents more influential keywords.

In addition to search terms, VOSviewer analyzed the keywords extracted from the titles and abstracts of 19,577 papers. Of these keywords, 127 terms that appeared more than 70 times were separated into 4 clusters. According to Figure 7A, Cluster 1 (in red, 44 items) was mainly about the research on mechanism of RSV. Cluster 2 (in green, 42 items) paid close attention to the study on the epidemiology and diagnosis of RSV. The analysis results indicated that the main target of RSV infection is infants and young children, and the clinical symptoms are similar to those of influenza. In addition, some researchers studied the effect of the COVID-19 epidemic on the epidemic characteristics of RSV. Cluster 3 (in blue, 21 items) studied RSV infection and immunity. Cluster 4 (in yellow, 20 items) was about the epidemiology, prevention, and disease burden of RSV. The size of the nodes involves occurrence frequency. In Figure 7B, VOSviewer classifies all keywords by colors based on the average publication year (APY). Among the displayed keywords, except for “rsv”(Cluster 3, APY: 2022.55) with 1,063 occurrence, the latest keyword was “influenza” (Cluster 2, APY: 2022.45) with 900 occurrence, followed by “sars-cov-2” (Cluster 2, APY: 2022.42) with 553 occurrence, “impact” (Cluster 2, APY: 2022.45) with 374 occurrence, and “prevention” (Cluster 3, APY: 2022.45) with 109 occurrence, and these were the most recent hot topics in this field. Many researchers are now paying more attention to the relationship between RSV and other respiratory viruses and its prevention. Compared with Figure 5, studying mixed infections with RSV and other microorganisms is the latest.

Additionally, Figure 7C shows that “respiratory syncytial virus,” “respiratory viruses,” “respiratory syncytial virus infection,” “respiratory syncytial virus fusion protein,” “alveolar macrophage,” “bovine respiratory disease,” “respiratory health,” “rice stripe virus,” “influenza virus,” and “mrna vaccine” have long received attention for research in RSV. In the analysis of the top 20 keywords with the strongest citation bursts (Figure 7D), the terms “infectious disease,” “sars coronavirus” “public health measures,” were the top 3 strength keywords, while the terms “release,” “load,” “molecular docking,” and “airway hyperresponsiveness” were the most recent trending keywords during the last 1 year. Figures 7A–D present the common theme is the study of the infection and epidemiological characteristics of RSV. In the future, more literature will study the basic research of RSV, and more researchers will be inclined to explore the pathogenesis and treatment mechanism and then investigate more possibilities for the prevention and therapy of RSV.

## Discussion

In recent years, the number of cases of RSV infection worldwide has been increasing yearly, attracting more attention from researchers. At present, bibliometric analysis is increasingly used in studying the current situation and trends in specific fields (Kokol et al., 2021; Zhu et al., 2021). This study is first bibliometric study of global research on RSV, adopts the bibliometric analysis method, and uses VOSviewer and Citespace software to explore the development trends and hotspots of RSV research from the WoSCC database. A total of 7,238 articles and reviews published between 2020 and 2024 were searched. Although the number of published papers has fluctuated slightly over the past decade, according to polynomial fitting curves, the overall trend of published articles is on the rise. Next, based on the frequency and co-occurrence relationship of keywords in each category, an in-depth analysis of literature under each category was conducted.

Publications on the study of RSV are scattered worldwide, but productivity in many countries is not high. In the past 5 years, the USA had the highest NPs (NP 2,278), far exceeding other countries/regions, indicating that the USA is a very prolific country in researching RSV. China ranked second (NP 1,524) and far exceeded the following countries. Although the Netherlands had a low NP (335), it had a higher H-index (48). Among the top 10 affiliations, most of them are in the USA (7), 2 are in England, and 1 is in France. Furthermore, 2 affiliations are from the USA, as well as Scotland and the Netherland. Unexpectedly, although China had a high output in the field of RSV, the top 10 research institutions and authors have not listed China. This observation indicates that Chinese scholars and institutions should make more efforts in improving the quality of papers in this field. Overall, American scholars have shown outstanding performance in the quantity and quality of their publications. The USA has the most outstanding institutions and experts, which helps explain why it has had such a remarkable influence on this topic over the past 5 years.

Moreover, the H-index and NC of the USA were much higher than those of other countries, indicating that it has conducted more in-depth research on this topic than other regions around the world and has good academic capabilities. The reason for this phenomenon may be that RSV was first isolated in 1955 by American scholars, and RSV has a disease burden similar to that of non-pandemic influenza A on the elderly and high-risk adults in the USA (Graham and Anderson, 2013). The recognition of the considerable disease burden caused by RSV has sparked interest in developing effective interventions and led to substantial investments from pharmaceutical companies and charitable organizations.

In addition, most of the top 10 journals with the highest output have high IFs (IF > 3), which indicates that the overall research on RSV is at a high level and has also triggered scholars interested in this topic to pay more attention to these journals. The “Viruses Basel,” “Journal of Infectious Diseases,” and “Frontiers in Immunology” made substantial contributions. The scientific directions and research topics covered by these journals are more relevant to scholars’ work, which is likely to encourage them to submit research to these journals. In summary, the journals listed in Table 5 are professional journals with high reputation and influence in the field of virology research. Therefore, scholars selecting these journals will be more likely to promote their ideals or viewpoints in this scientific field, enabling them to discuss and exchange their ideas with peers, to improve their academic level and scientific ability. This development also prompts scholars interested in this topic to read these publications more carefully.

From Figure 6D, it can be seen that in the top 20 references with the strongest citation bursts, survey reports on the prevalence of RSV account for more than half of the total. And these references were mainly published between 2016 and 2021. In Figures 6A–C, based on cluster analysis, the timeline views of commonly used references and keywords show that the hot topics that have been widely studied by researchers are “protein vaccines,” “structural insights,” and “respiratory virus co infections,” which have also become research hotspots that researchers have begun to pay attention to in recent years. It means that researchers’ focus has begun to shift from the epidemiology of RSV in the past to prevention and treatment now. Similarly, Hammitt, MD et al. evaluated the efficacy and safety of nirsevimab, and their results indicated that a single injecting nirsevimab before the RSV season can protect healthy late-preterm and term infants from medically attended RSV-related lower respiratory tract infections (Hammitt et al., 2022). Simoes, EAF et al. reported two recombinant monoclonal antibodies currently undergoing randomized clinical trials, namely, nirsevimab and suptavumab, which can be used to protect healthy infants born at term or preterm from RSV infection (Griffin et al., 2020; Simoes et al., 2021). In 2023, Mazur NI et al. published a review on “*Lancet Infectious Diseases,”* which provided an overview of RSV vaccines and monoclonal antibodies in clinical development highlighting different target populations, antigens, and trial results (Mazur et al., 2023). Based on this speculation, monoclonal antibodies may become a key focus for guarding against RSV infection in future. Previously, most researchers focused on infant infections. However, reference analysis in Figure 6C shows that more people are now shifting their attention to RSV infections and the safety of vaccination for the older adults (Ackerson et al., 2019; Korsten et al., 2021). Moreover, the research on COVID-19 focuses on developing vaccines and treatment strategies targeting it based on RSV research (Hsieh et al., 2020; Taylor et al., 2021). Overall, analysis of co-cited literature indicates that in the past 5 years, researchers had attached great importance to the prevention and treatment of RSV.

With the progress of RSV research, some emerging research fields are gradually becoming research topics of interest to researchers. As showed in Figure 7A, a co-occurrence network diagram was created by evaluating the keywords in all indexed publications, and Figure 7B shows “sars-cov-2” and “influenza virus” are the latest hot words in the field of RSV research, which indicates that more researchers have begun to pay attention to the mixed infection of microorganisms that cause respiratory diseases. Because they are all viruses that can cause respiratory diseases, SARS-COV-2 has inevitably become a popular word in RSV research in the past 5 years. Many researchers have shown that the epidemiology of RSV has undergone (Sullivan et al., 2020; Stera et al., 2021). The reason for this phenomenon may be related to changes in our lifestyles and vaccination at that time. Furthermore, currently, given the lack of effective prevention and treatment strategies for many viral diseases, the development of vaccines and antiviral drugs has always been a challenge that must be urgently addressed. Therefore, drugs and measures that effectively combat RSV infection, such as “resveratrol,” “vaccine,” and “monoclonal antibody,” all appear in the hot-word analysis. Resveratrol has antioxidant, anti-inflammatory, and antimicrobial properties. Many studies have shown that it exhibits good antiviral activity against various viruses that cause severe respiratory infections, such as influenza virus, RSV, and SARS-cov-2 (Xiong et al., 2024; Filardo et al., 2020). In recent years, many scholars have shifted their attention to the research on non-pharmacological intervention (NPI). They have compared and analyzed the roles of various respiratory viruses, including NPI, in their transmission. The results show that developing targeted NPI can effectively respond to the spread of viruses (Ye and Liu, 2022). These studies can provide reference for effective response to emerging infectious diseases in the future.

Furthermore, through analysis of the top 20 keywords with the strongest citation bursts, we identified distinct research priorities and collaborative patterns among high-output countries in RSV research. The USA independently published three high-impact studies that addressed RSV vaccine developmentvira (Higgins et al., 2016), bronchiolitis in children (Meissner, 2016), and delayed seasonal RSV surges (Agha and Avner, 2021), demonstrating its core technological competencies and multidisciplinary research capacity. China contributed two significant publications focusing on clinical characteristics and mortality risk factors in COVID-19 patients, highlighting its specialized expertise in viral epidemiology (Zhou et al., 2020; Huang et al., 2020). The United Kingdom produced two studies that examined immune responses to RSV infection (Openshaw et al., 2017; Russell et al., 2017), while Canada published one comprehensive review that systematically analyzed RSV detection, prevention, and treatment strategies (Cane, 2001). Internationally collaborative publications accounted for 60% (12/20) of these high-impact papers, with joint research efforts primarily concentrating on RSV clinical case analyses, epidemiological investigations, viral characterization and classification, as well as advancements in vaccine and monoclonal antibody development (Liu et al., 2016; Rima et al., 2017; Shi et al., 2017; Mazur et al., 2023; Griffiths et al., 2017; Stein et al., 2017; de Steenhuijsen Piters et al., 2016; Bont et al., 2016; Scheltema et al., 2017; Afonso et al., 2016; Florin et al., 2017; Jartti and Gern, 2017; Acosta et al., 2015). The USA not only independently conducted a landmark study on RSV vaccines but also led several key collaborative publications, including one influential review that outlined progress in RSV vaccine and monoclonal antibody research and provided important guidance for future research directions. This dual-track model combining independent innovation with international collaboration yielded significant insights. The U.S. leadership in vaccine development served as a technological driver for global research, while multinational collaborative studies substantially broadened the research scope of RSV, encompassing clinical, epidemiological, and fundamental scientific aspects, while accelerating breakthroughs in critical areas including vaccine development. Vaccines and monoclonal antibodies persisted as the central focus of RSV research. Enhanced international cooperation, particularly through resource integration among high-productivity nations, demonstrated potential to further improve the efficiency of RSV vaccine research and development.

Through the cluster analysis in Figures 6, 7, we have found that research on RSV co-infections, pathogenic mechanism, antiviral immune responses, and vaccine development have become hotspots over the past 5 years. Lansbury L and Swets MC et al. assessed the burden of bacterial co-infections in COVID-19 patients and found that the combined proportion of viral co-infections was 7 and 8.4%, with RSV and influenza A being the most common (Lansbury et al., 2020; Swets et al., 2022). Griffiths CD et al. revealed that insulin-like growth factor 1 receptor (IGF1R) was the entry receptor for RSV, and the binding of the prefusion RSV-F glycoprotein to the IGF1R triggered the activation of protein kinase C zeta (PKCζ). Through this signaling mechanism, nucleolin moved from the nuclei of cells to the plasma membrane and associated with RSV-F on viral particles (Griffiths et al., 2020; Rima et al., 2017). Hu M et al. used high-resolution quantitative imaging, bioenergetics measurements, and mitochondrial membrane potential and redox-sensitive dyes for the first time to define the impact of RSV on host mitochondria, identifying the unique ability of RSV to sequester host cell mitochondria to facilitate viral infection (Hu et al., 2019). Akagawa M et al. investigated the molecular interactions between prefusion RSV-F and the cell receptor complex composed of Toll-like receptor 4 (TLR4) and myeloid differentiation factor 2 (MD-2), revealed that conformational changes in the F protein, which increased its binding affinity, may facilitated the formation of syncytia in RSV-infected cells, thereby enhancing the understanding of RSV infectivity and pathogenicity (Akagawa et al., 2022). During RSV infection, the host’s innate and adaptive immune responses were induced. RSV infected the epithelial cells of the respiratory tract, and alveolar macrophages actively participated in viral detection and initiate rapid antiviral responses (Panahipoor Javaherdehi et al., 2024). Innate immune cells controlled viral replication and induced immune pathology through intracellular signaling pathways composed of pattern recognition receptors (PRRs), adaptors, kinases, and transcription factors. Complex processes of ubiquitination and deubiquitination played regulatory roles in the transmission of these signaling pathways (Martin-Vicente et al., 2022). Alveolar macrophages influenced the adaptive immune response by regulating the activation of T lymphocytes and the secretion of key cytokines (Panahipoor Javaherdehi et al., 2024). Luangrath MA et al. found that RSV-specific CD4+ and CD8+ TRM cells were established in the lungs and decreased over time after the initial infection. CD8 TRM cells demonstrated protective immunity, suggesting that inducing lung-resident TRM cells should be a key focus in RSV vaccine development (Luangrath et al., 2021). The RSV surface fusion glycoprotein has become a new target for preventive interventions against RSV, with 19 candidate vaccines and monoclonal antibodies (mAbs) developed using four approaches, particle-based, live-attenuated or chimeric, subunit, and vector-based (Mazur et al., 2018). Nirsevimab, a monoclonal antibody targeting the RSV fusion protein, could protect healthy late preterm and term infants from medically attended RSV associated lower respiratory tract infections with a single injection (Hammitt et al., 2022). Sequence analysis of RSV F/G glycoproteins by Dar HA et al. identified strongly associated CD8+/CD4+ T cell epitopes with HLA alleles. A resulting multi-epitope vaccine combining these epitopes with adjuvants may elicit dual protective immunity against RSV (Dar et al., 2022).

Based on the bibliometric analysis of RSV literature, this study systematically combed through the developmental trajectory of RSV research, deeply dissected the evolutionary process, current research status, and future trends in this field. Early research mainly focused on the clinical epidemiological characteristics of RSV, including basic studies such as disease burden assessment, transmission dynamics, seasonal epidemic patterns, and viral subtype distribution. Recently, the research focus has shifted to the elucidation of pathogenic mechanisms at the molecular level, such as: (1) the crucial role of conformational changes in RSV F protein and M protein during viral entry into the host; (2) the host’s innate immune response and antiviral defense mechanisms; (3) breakthroughs in vaccine development technology based on structural biology; (4) the establishment of genomics and precision monitoring systems driven by high-throughput sequencing. Future research directions may cover the following key areas: (1) Antiviral treatment strategies, including the development of small-molecule inhibitors, optimization of broad-spectrum antiviral drugs, and resistance monitoring; (2) Passive immunotherapy, such as clinical optimization and expansion of the target population for long-acting monoclonal antibodies; (3) New vaccine technologies, innovative design based on mRNA, virus-like particles (VLP), or T-cell vaccines; (4) Immune regulation and precision medicine, optimization of maternal vaccination strategies to enhance mother-to-child immune protection, and personalized immune interventions; (5) Global health and public health policy, improving vaccine accessibility, perfecting resistance monitoring networks, and formulating regional prevention and control strategies.

Analysis of highly cited publications revealed that international collaborative efforts were predominantly concentrated on RSV vaccine and monoclonal antibody development, which aligned with future research priorities including novel vaccine technologies and antiviral therapeutic strategies. This synergy demonstrated that multinational cooperation was directly advancing key future research areas. The collaborative studies not only accelerated breakthroughs in existing vaccine technologies, such as the application of mRNA vaccine platforms, but also addressed critical gaps in vaccine accessibility through resource sharing-establishing a closed loop connection with future global health policy research directions. However, these collaborations had not yet adequately covered emerging fields like immunomodulatory mechanisms and precision medicine applications. This finding suggested that enhanced transnational cooperation between basic research and clinical translation was required, along with the establishment of international multicenter research networks targeting RSV-specific immune responses.

The data collection period of this study coincided with the COVID-19 pandemic, during which many research institutions and journals prioritized COVID-19-related research and accelerated its publication. This may resulted in disproportionate emphasis on COVID-19 findings while potentially marginalizing research on other viral respiratory infections, including RSV. Such publication bias could compromise the representativeness and timeliness of our findings. Future studies could address this limitation by comparing publication patterns before, during, and after the pandemic to better assess its impact on research outcomes.

Bibliometric analysis and literature visualization can enhance the understanding of the development trends and hotspots in this field. However, this study has some limitations. First, although the WoSCC data retrieved can represent a large amount of information, excluding some literature from these two databases should be considered. Moreover, the design of the retrieval strategy is beneficial for the matching accuracy of the retrieval, and the NCs may be underestimated. Second, this article uses CiteSpace and VOSviewer for bibliometric analysis, and only analyzing the main conclusions rather than full-text analysis may result in the exclusion of some information. Therefore, it cannot completely replace full-text search. Finally, due to the exclusion of some newly published excellent papers with low NC, this study has a certain degree of lag.

## Conclusion

This study focused on revealing the development trends and research hotspots in the field of RSV over the past 5 years. Clinical case analysis and disease burden of RSV, epidemiology and transmission patterns, pathogenic mechanisms, antiviral immune responses, antiviral treatments, and vaccine development were all identified as current research hotspots. International collaboration played a significant role in advancing breakthroughs in existing vaccine technologies, and through resource sharing, it further clarified the future research directions for global health strategies.

## Data Availability

The datasets presented in this study can be found in online repositories. The names of the repository/repositories and accession number(s) can be found in the article/Supplementary material.

## Footnotes

^1^ http://www.bioinformatics.com.cn/

## Supplementary material

SUPPLEMENTARY MATERIAL S1 Top 10 countries in terms of annual publications on RSV research from 2020 to 2024.

SUPPLEMENTARY MATERIAL S2 Top 10 research productive research area on RSV.
