# Supplementary material for: Research hotspots and global trends in respiratory syncytial virus over past five years
Source: Front Microbiol. 2025 Aug 4;16:1599093. doi: 10.3389/fmicb.2025.1599093 (PMC12358382; doi:10.3389/fmicb.2025.1599093)
Supplement: SUPPLEMENTARY MATERIAL S1 — Top 10 countries in terms of annual publications on RSV research from 2020 to 2024. [file Supplementary_file_1.docx]

Supplementary Material S1 | Top 10 countries/regions in terms of annual publications on RSV research from 2020 to 2024.

| **Country** | **2020** | **2021** | **2022** | **2023** | **2024** |
| --- | --- | --- | --- | --- | --- |
| USA | 451 | 483 | 379 | 438 | 527 |
| CHINA | 238 | 302 | 311 | 307 | 366 |
| ENGLAND | 110 | 108 | 93 | 100 | 116 |
| ITALY | 91 | 83 | 87 | 99 | 115 |
| FRANCE | 67 | 86 | 71 | 78 | 108 |
| SPAIN | 57 | 72 | 62 | 70 | 111 |
| AUSTRALIA | 74 | 77 | 67 | 69 | 60 |
| NETHERLANDS | 61 | 65 | 65 | 58 | 86 |
| CANADA | 58 | 78 | 48 | 66 | 62 |
| GERMANY | 49 | 58 | 65 | 63 | 70 |

Supplementary Material S2 | Top 10 research productive research area on RSV.

| **Rank** | **Research Area** | **2020** | **2021** | **2022** | **2023** | **2024** |
| --- | --- | --- | --- | --- | --- | --- |
| 1 | Immunology | 251 | 256 | 245 | 226 | 293 |
| 2 | Infectious Diseases | 195 | 253 | 215 | 236 | 316 |
| 3 | Microbiology | 154 | 195 | 177 | 188 | 212 |
| 4 | Virology | 19 | 189 | 152 | 184 | 224 |
| 5 | Pediatrics | 121 | 148 | 128 | 152 | 185 |
| 6 | Pharmacology Pharmacy | 102 | 134 | 120 | 100 | 155 |
| 7 | Biochemistry Molecular Biology | 99 | 111 | 116 | 116 | 95 |
| 8 | Research Experimental Medicine | 104 | 105 | 74 | 92 | 121 |
| 9 | Science Technology Other Topics | 79 | 3 | 96 | 88 | 109 |
| 10 | General Internal Medicine | 52 | 80 | 71 | 75 | 98 |
